# Non-readmission decisions in the intensive care unit under French rules: A nationwide survey of practices

**DOI:** 10.1371/journal.pone.0205689

**Published:** 2018-10-18

**Authors:** Jean-Philippe Rigaud, Mikhael Giabicani, Nicolas Meunier-Beillard, Fiona Ecarnot, Marion Beuzelin, Antoine Marchalot, Auguste Dargent, Jean-Pierre Quenot

**Affiliations:** 1 Service de Médecine Intensive Réanimation, Centre Hospitalier de Dieppe, Dieppe, France; 2 Service de Médecine Intensive Réanimation, Université de Bourgogne Franche Comté, CHU de Dijon, Dijon, France; 3 UMR 7366 CNRS, Université de Bourgogne Franche Comté, Centre Georges Chevrier, Dijon, France; 4 EA3920, Department of Cardiology, University Hospital Besancon, and University of Burgundy Franche Comté, Besançon, France; 5 Lipness Team, INSERM, UMR 1231, Université de Bourgogne Franche Comté, Dijon, France; 6 INSERM CIC 1432, Faculté de médecine de Dijon, Université de Bourgogne Franche Comté, Dijon, France; Azienda Ospedaliero Universitaria Careggi, ITALY

## Abstract

**Purpose:**

We investigated, using a multicentre survey of practices in France, the practices of ICU physicians concerning the decision not to readmit to the ICU, in light of current legislation.

**Materials and methods:**

Multicentre survey of practices among French ICU physicians via electronic questionnaire in January 2016. Questions related to respondents’ practices regarding re-admission of patients to the ICU and how these decisions were made. Criteria were evaluated by the health care professionals as regards importance for non-readmission.

**Results:**

In total, 167 physicians agreed to participate, of whom 165 (99%) actually returned a completed questionnaire from 58 ICUs. Forty-five percent were aged <35 years, 74% were full-time physicians. The findings show that decisions for non-readmission are taken at the end of the patient’s stay (87%), using a collegial decision-making procedure (89% of cases); 93% reported that this decision was noted in the patient’s medical file. While 73% indicated that the family/relatives were informed of non-readmission decisions, only 29% reported informing the patient, and 91% considered that non-readmission decisions are an integral part of the French legislative framework.

**Conclusion:**

This study shows that decisions not to re-admit a patient to the ICU need to be formally materialized, and anticipated by involving the patient and family in the discussions, as well as the other healthcare providers that usually care for the patient. The optimal time to undertake these conversations is likely best decided on a case-by-case basis according to each patient’s individual characteristics.

## Introduction

Decisions to limit or withdraw treatment in the intensive care unit (ICU) usually concern patients in whom every possible curative therapeutic option has been attempted, and/or in whom pursuing treatment would be unreasonable in view of their current clinical state and likely outcome [1–3]. In France, as in numerous other countries [3], decisions to limit or withdraw treatment are made within a strict legislative framework that involves a collegial decision-making procedure [4]. The collegial procedure must take into account the patients’ wishes, if they are known or have been recorded. When the patient’s wishes are not documented, the opinion of the family and/or surrogate must be sought. In addition, the opinion of the caregiving team, the patient’s referring physicians (if any), and any advance directives formulated by the patient must also be taken into consideration in what is truly a collective and multidisciplinary approach [5]. Once a decision has been reached, life-sustaining therapies are limited or withdrawn in conditions that have previously been evaluated [6, 7], and the patient can be oriented towards palliative care [4, 5].

However, due to the severity of disease, a complex medical history, numerous comorbidities, a future level of autonomy that is anticipated to be poor, or because of unfavourable outcome in the ICU, for some patients discharged from the ICU, a decision is made not to re-admit to intensive care if the question should arise again in the future. The main concern of the physicians in such cases is to re-orient the patient’s healthcare project in order to avoid “unreasonable therapeutic obstinacy”, but they must also be fully certain that they are not depriving the patient of access to care resources that could keep him/her alive, and which it is our moral duty to dispense. This decision should not be the sole responsibility of the ICU physician, but should also involve the physicians that know the patient best (e.g. general practitioner (GP), specialists…) in the framework of discussions between professionals [8, 9]. While the ICU physician is likely the physician that best knows the potential benefits and adverse effects of intensive care techniques, he/she is rarely the one who knows the patient best, especially in the context of chronic disease [10–12].

Currently, decisions “not to re-admit” are generally not made through a collegial procedure, even though such a decision could legitimately be considered as a limitation on access to therapeutic resources. Data in the literature are sparse on this topic and mostly stem from studies evaluating the prognostic impact of the timing of discharge from the ICU [13, 14] or the determinants of mortality after a stay in the ICU [15]. The frequency of decisions not to re-admit to the ICU varies from 6 to 11% depending on the study [13–15], with in-hospital mortality exceeding 50% in these patients [14, 15]. To the best of our knowledge, the processes leading to a decision not to re-admit a patient being taken before the patient’s discharge from the ICU have never been specifically investigated.

In this context, our goal was to describe the practices of ICU physicians in France concerning the decision not to readmit to the ICU, in light of current legislation.

## Methods

### Study design and questionnaire

We performed a survey of practices among physicians working in French ICUs using an electronic questionnaire sent in January 2016 to a list of physicians (N = 250) compiled for use in two previous studies [16, 17]. A questionnaire was developed comprising 21 questions about respondents’ practices in terms of re-admission of patients to the ICU during the same hospital stay and how these decisions were made. The questionnaire was constructed by 2 ICU specialists and a sociologist. The empirical data used to build the questionnaire items were obtained from a preliminary qualitative study among 18 physicians working in the ICU setting. This exploratory phase used *in situ* observations and semi-structured interviews (open-ended questions in one-to-one interviews) to identify factors that were potentially important for physicians to decide on non-readmission to the ICU.

Interviews were conducted until saturation of concepts; that is, interviews were stopped when the last interview yielded no new information likely to add to the empirical data already recorded. After these interviews, the items that came out of the discourse were rephrased to achieve maximum readability (using the Flesch readability test and the Flesch-Kincaid grade level). Next, the expert panel method was applied to reduce the number of items present in the final questionnaire as previously described [18].

The questionnaire is provided in Table 1.

**Table 1:** Questionnaire regarding decisions not to re-admit a patient to the intensive care unit.

No.	Question	Response options
1	What is the average number of admissions per month in your intensive care unit (ICU)?	<50 50–75 75–100 >100
2	Please give an estimate of how many patients per month are the subject of a decision not to re-admit to the ICU?	<5 5–10 10–15 >15
3	Is the decision not to re-admit a patient to the ICU taken at the end of the patient’s initial stay in the ICU?	Yes, all the time Yes, most of the time Sometimes Rarely Never
4	Are decisions not to readmit made using a collegial decision-making procedure in your unit?	Yes, all the time Yes, most of the time Sometimes Rarely Never
5	Are decisions not to readmit mentioned in the discharge letter?	Yes, all the time Yes, most of the time Sometimes Rarely Never
6	Are decisions not to readmit recorded in a registry in your unit?	Yes No
7	Is the unit that will receive the patient after discharge from the ICU informed of (without being involved in) the decision not to readmit?	Yes, all the time Yes, most of the time Sometimes Rarely Never
8	Is the unit that will receive the patient after discharge from the ICU involved in the decision not to readmit?	Yes, all the time Yes, most of the time Sometimes Rarely Never
9	Does an independent outside consultant participate (if necessary) in the decision not to readmit?	Yes, all the time Yes, most of the time Sometimes Rarely Never
10	Does the patient participate in the discussions leading to a decision not to readmit to intensive care?	Yes, all the time Yes, most of the time Sometimes Rarely Never
11	Does the patient's family and/or surrogate participate in the discussions leading to a decision not to readmit to intensive care?	Yes, all the time Yes, most of the time Sometimes Rarely Never
12	Is the patient informed about the decision not to readmit?	Yes, all the time Yes, most of the time Sometimes Rarely Never
13	Is the patient's family and/or surrogate informed about the decision not to readmit?	Yes, all the time Yes, most of the time Sometimes Rarely Never
14	Is the GP or referring specialist involved in the collegial decision-making procedure?	Yes, all the time Yes, most of the time Sometimes Rarely Never
15	Is the GP or referring specialist informed about the decision not to readmit?	Yes, all the time Yes, most of the time Sometimes Rarely Never
16	Are the criteria justifying the decision not to readmit noted in the patient's medical file?	Yes, all the time Yes, most of the time Sometimes Rarely Never
17	Are the criteria used to decide not to readmit to the ICU the same as those applied to not to admit a patient to the ICU a first time?	Yes No If no, please explain
18	If a collegial decision-making procedure is used to decide not to readmit a patient to the ICU, are there circumstances in which you may fail to respect that decision?	Yes No Please explain
19	Do you distinguish between a request to readmit a patient to the ICU for an intercurrent acute episode, and a request to readmit a patient for worsening of the underlying chronic disease?	Yes No Please explain
20	When a decision not to readmit is made, do you plan palliative care?	Yes, all the time Yes, most of the time Sometimes Rarely Never
21	In your opinion, is non-readmission to the ICU covered by current end-of-life legislation in France?	Yes No If no, please explain

### Survey definitions

The non-readmission patient was defined as a patient hospitalised in the ICU and for whom a decision not to re-admit to the ICU was taken during the same hospital stay.

Each of the criteria was to be evaluated by the health care professionals as regards its importance for non-readmission. Fourteen responses were on a scale of frequency (yes, all of the time; yes, most of the time; sometimes; rarely; never). Five responses were yes/no questions with room for a commentary. For two items, the respondent had to tick the appropriate option from among a range of percentages, and finally, there were 7 questions relating to the respondent’s age, gender, job title (full-time staff physician, professor, junior physician), number of years’ experience in the ICU, practice location (academic or non-academic ICU in France), type of ICU (mixed, medical, surgical), number of admissions per month and per ICU, number of non-readmissions per month and per ICU. The number of non-readmissions per month and per ICU was estimated based on data recorded from the discharge letters concerning patients in the unit over the previous 3 months.

A letter detailing the protocol was sent to all physicians together with the questionnaire. All questionnaires were anonymous. This methodology has previously been described elsewhere [17, 19].

### Statistical analysis

Qualitative variables are expressed as numbers (percentages) and were compared using the Chi square or Fisher’s exact test as appropriate. It should be noted that the response categories “yes, all the time” and “yes, most of the time” were merged, as were the categories “rarely” and “never”.

Associations between physician (number of years’ experience) and ICU characteristics (type of unit, number of beds) and re-admission and communication practices (documentation, decision-making, information to patients, families, other providers) were explored by univariate analysis.

A p-value of <0.05 was considered statistically significant. All analyses were performed using SAS version 9.1 (SAS institute Inc., Cary, NS, USA).

## Results

### Characteristics of the respondents

In total, 250 physicians were contacted, and 167 replied that they agreed to participate, and 165 (99%) actually returned a completed questionnaire, yielding a response rate of 67%. At least one physician responded from all 58 ICUs in France that were contacted. The median number of respondents per unit was 2 (interquartile range 1–4). The characteristics of the respondents are shown in Table 2.

**Table 2:** Characteristics of the intensive care units and intensive care physicians who responded to the questionnaire.

	Physicians (n = 165)
Age (years), n (%)	
<35	75 (45)
35–50	32 (19)
>50	58 (35)
Sex, male n (%)	126 (76)
Grade of respondent, n (%)	
Full-time staff physician	122 (74)
Professor	28 (17)
Junior physician	15 (9)
Number of years of ICU practice, n (%)	
<5	28 (17)
5–20	79 (48)
>20	58 (35)
Practice location, n (%)	
Academic	92 (56)
Non-academic	73 (44)
Type of critical care unit, n (%)	
Mixed	86 (52)
Medical	59 (36)
Surgical	20 (12)
Number of admissions/month/ICU, n (%)	
<50	27 (16)
50–75	65 (39)
75–100	40 (24)
>100	33 (20)
Estimated number of non-readmissions/month/ICU, n (%)	
<5	89 (54)
5–10	65 (39)
10–15	8 (5)
>15	3 (2)

### Responses to questionnaire

**Fig 1:**
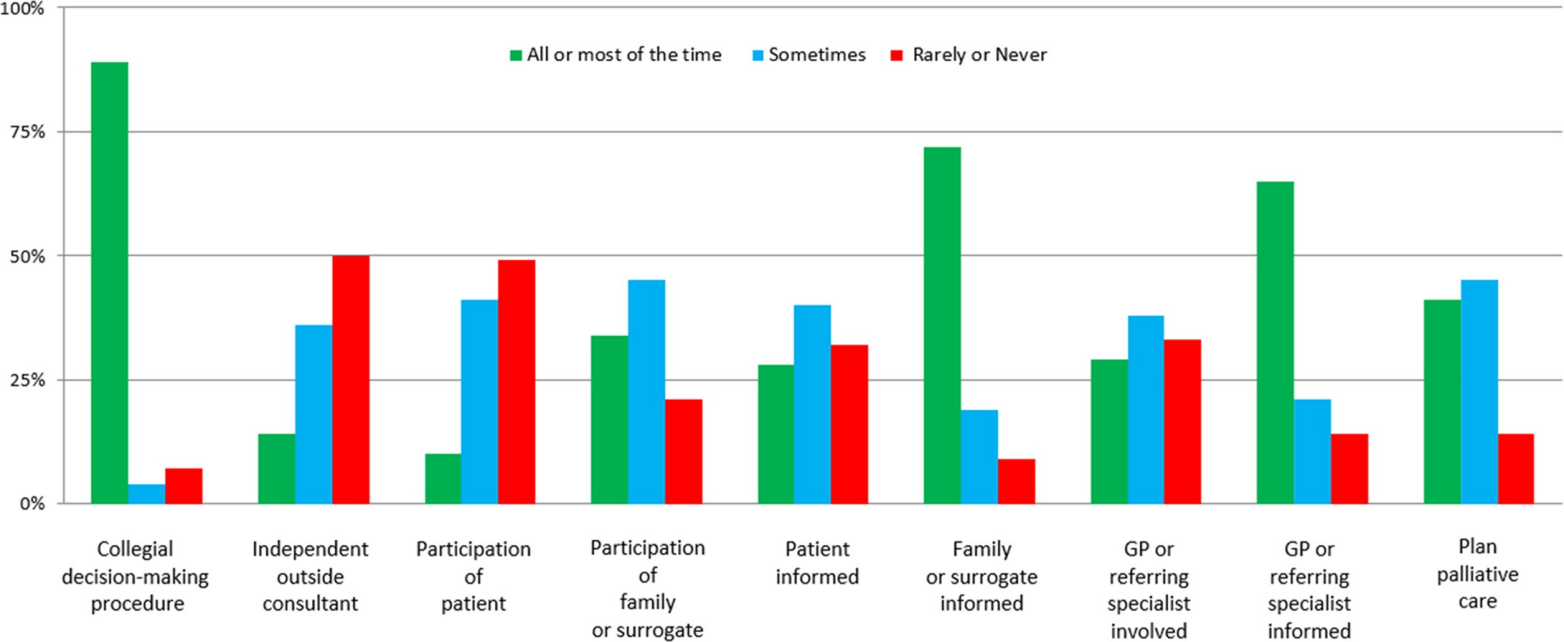
Responses to the questionnaire regarding how re-admission decisions are taken in the intensive care unit (N = 165 respondents). GP, general practitioner.

The details of the responses to the questionnaire are summarised in Fig 1. Detailed results are available in the supporting information (S1 File). The findings show that decisions for non-readmission are reportedly taken at the end of the patient’s stay (87%), and physicians report taking these decisions using a collegial decision-making procedure (89%), and 93% reported that this decision was then noted in the patient’s medical file. Respondents stated that they informed the unit where the patient was being transferred from the ICU about the non-readmission decision in 83% of cases, although they indicated that physicians from the receiving unit were involved in the decision in only 30% of cases. While 73% indicated that the patient’s family/relatives were informed of the decision not to re-admit, only 29% reported informing the patient.

Regarding the participants in the decision-making process, respondents reported that those involved included the family and/or relatives, an outside consultant physician, a general practitioner (GP) or another specialist who regular cared for the patient in respectively 10%, 34%, 14% and 29%. Conversely, 85% responded that the information regarding the non-readmission decision was communicated to the other physicians (GP or specialist) in charge of the patient’s usual care, albeit without details of how the information was given. Although the criteria justifying the decision not to re-admit were reported to be documented in the medical file by 91% of respondents, a decision to initiate palliative care was reportedly taken in only 41%.

Overall, 96% of respondents stated that they did not keep a list of “patients who are not to be re-admitted”. Similarly, 61% of respondents concurred that the criteria for non-readmission are largely the same as those leading to a decision to limit or withdraw therapy. Conversely, 92% of those who completed the survey said that they made a clear distinction between a request for re-admission to the ICU due to an intercurrent acute episode, and a request for re-admission due to deterioration of a chronic disease.

Finally, the majority of respondents (91%) considered that non-readmission decisions are an integral part of the French legislative framework regarding decisions to limit or withdraw therapy.

### Univariate analysis

By univariate analysis, the number of years’ experience of the respondents was significantly associated with improved tracability in the patient file (p = 0.03), and with a more frequent propensity to inform the patient (p = 0.04). Similarly, there was a significant relationship between the type of critical unit (mixed, medical, surgical) in which the respondent worked, and the propensity to inform the patient’s family of non-readmission decisions (p = 0.01).

## Discussion

To the best of our knowledge, this is the first study to specifically study the conditions in which non-readmission decisions are taken for patients in the ICU. The main findings show that decisions for non-readmission are taken at the end of the patient’s stay (87%), using a collegial decision-making procedure (89% of cases); 93% reported that this decision was noted in the patient’s medical file. While 73% indicated that the family/relatives were informed of non-readmission decisions, only 29% reported informing the patient, and 91% considered that non-readmission decisions are an integral part of the French legislative framework.

ICU admission or readmission has traditionally been reserved for patients with reversible medical conditions and a reasonable prospect of substantial recovery [20]. Re-admission of a patient to the ICU during a same hospital stay occurs in 5 to 10% of patients, and these patients have a two- to three-fold increase in the risk of death as compared to patients with similar initial severity of disease, but who do not require ICU re-admission [21]. The risk factors for re-admission to the ICU, as well as the resources that are mobilized to reduce morbidity and mortality in these patients have been well described [22–25].

In the literature, different approaches have been proposed to address the thorny issue of ICU re-admission, such as advance care planning (ACP) in agreement with the patient and his/her family or relatives, ethics consultations and palliative care consultations [26–30]. However, despite all these efforts, it remains clear that the ICU physician must be the arbiter of decisions regarding re-admission or non-re-admission of a patient to the ICU [1, 9]. Several arguments can be advanced to justify this position [31]. Firstly, the intensivist best knows the patient’s healthcare pathway in the ICU. Second, the intensivist is also best placed to evaluate the patient’s prognosis according to the existence of organ failure. Third, the intensivist is again the best placed to explain to the patient and/or family the aims, limitations and consequences of re-admission to the ICU.

In addition, a number of other factors may also come into play in the decision, such as physical and/or psychological distress in the patient and/or family; inappropriateness of the unit where the patient is currently hospitalized, lack of information in the medical files regarding end-of-life preferences or lack of knowledge of the patient’s choices or the family’s wishes. All these issues represent difficulties that intensive care physicians regularly have to face in emergency situations [31–34]

Ninety percent of the physicians surveyed in our study reported that they addressed the question of non-readmission with around 10% of their ICU patients. This is obviously a frequent issue that poses the ethical dilemma of the limitation on access to care resources likely to keep the patient alive. In the study by Azoulay et al [15], non-readmission was significantly associated with in-hospital mortality after an initial stay in the ICU, with an odds ratio of 9.64. The authors justified this limitation on access to care by the severity of the underlying chronic disease and of the acute episode that prompted the initial ICU stay, as well as by the persistence of organ failure. Other authors have reported similar reasons for non-readmission [6, 13, 14, 35, 36] that are line with international recommendations for end-of-life care in the ICU [3, 20].

Our survey of practices brings to light some new aspects regarding the ways in which decisions for non-readmission to the ICU come to be taken. The collegial decision-making procedure seems to be effective for 90% when decisions are being made for patients who are unable to express themselves or who are decisionally incapacitated, which is fully in line with French legislation in this regard [4]. Conversely, only 29% reported informing the patients, whereas 73% reported that they informed the family. This could be partially explained by the fact that patients who are discharged from the ICU are generally not well enough to be able to receive this specific information, not only because of their health state but also because of the anxiety and stress caused by the ICU stay [36, 37]. Indeed, the patient’s family is also often affected by this stress, in a phenomenon that has been termed “post-intensive care syndrome” [38].

The question of when to make a decision not to re-admit a patient to the ICU is crucial, and two overriding frameworks can nonetheless be proposed that would likely cover the vast majority of circumstances.

First is when the clinical course and prognosis seem to be unfavourable in the short term, and then the decision can be made at the end of the patient’s ICU stay in a collective and interdisciplinary approach [10]. This formal meeting should involve the patient where possible, either directly (if the patient is competent) or via the family and/or surrogate [39], in order to take account of the choices and preferences expressed by the patient [40]. Other referring or treating physicians should also be involved where possible in this process to make their contribution to formally establish and ratify the new healthcare project. Indeed, the results of our survey show that few other contributors (particularly medical staff) from outside of the ICU are involved in decisions not to re-admit.

The second option is to make the decision for non-readmission after discharge from the ICU, regardless of whether the patient is still in hospital or not, as a means of anticipating the future occurrence of another acute episode into advance care planning [41]. The ICU physician could participate in this decision as an outside consultant, bringing the wealth of experience gained in intensive care medicine to the table to inform the decision, as well as their expert knowledge of the patient’s prior healthcare history in the ICU [42, 43]. In this situation, the intensivist has the knowledge to explain exactly what a new admission to ICU would entail given the patient’s health state, and what can be proposed in terms of life-sustaining therapies, and what it is reasonable to do (or not). In this regard, studies testing palliative care interventions or ethics consultations [30, 44] or a rapid response team in nosocomial end-of-life [31] have found a reduction in the number of ICU admissions, with greater respect of the patient’s wishes [45], although all these studies investigated the question of ICU admission and not “non-admission”.

### Study limitations

Our study suffers from some limitations that deserve to be underlined. Firstly, this was a survey of practices in France, and therefore, may not be amenable to generalisation to other countries or cultures. Secondly, the characteristics of the respondents recorded in this survey do not allow more detailed analysis of the potential influence of other factors, such as cultural or religious beliefs, in the decision-making process. Thirdly, nurses and nurses’ aides were not invited to participate in this study. Their opinions would have been interesting, as they are often deeply implicated in decision-making surrounding limitation and withdrawal of therapy in the ICU setting. Fourth, we cannot exclude the possibility that our questionnaire was not exhaustive and that other, unmeasured confounders were not taken into account.

Conversely, our study also presents several strong points. Firstly, the response rate was satisfactory since the majority of the participants have previously worked with our Department on other research projects. Secondly, the questionnaire was developed using methodologically robust qualitative methods based on semi-directive interviews, thus ensuring that the main issues arising in this context were identified, and addressed with relevant questions in the survey. Thirdly, as far as we know, this is the first study to specifically investigate the decision-making process regarding non-readmission to the ICU.

### Conclusion

This study shows that decisions not to re-admit a patient to the ICU need to be formally materialized, and anticipated by involving the patient and family in the discussions, as well as the other healthcare providers that usually care for the patient. The optimal time to undertake these conversations is likely best decided on a case-by-case basis according to each patient’s individual characteristics. The issue of ICU re-admission needs to be anticipated during the initial ICU stay to allow a time for reflection that is commensurate with the importance and the consequences of the decision.

## Supporting information

S1 File Supporting tables. Tables detailing responses to the questionnaire regarding decisions not to re-admit a patient to the intensive care unit, and percentage of responses for yes/no questions on the study questionnaire.
(DOC)

S2 File Detailed study results. The detailed results of the study are available in the supporting file.
(XLS)

S3 File COREQ checklist. The completed COREQ checklist is provided in the supporting file.
(DOCX)
