# Supplementary material for: Non-readmission decisions in the intensive care unit under French rules: A nationwide survey of practices
Source: PLoS One. 2018 Oct 18;13(10):e0205689. doi: 10.1371/journal.pone.0205689 (PMC6193659; doi:10.1371/journal.pone.0205689)
Supplement: S1 File — Tables detailing responses to the questionnaire regarding decisions not to re-admit a patient to the intensive care unit, and percentage of responses for yes/no questions on the study questionnaire. (DOC) [file pone.0205689.s001.doc]

**Non-readmission decisions in the intensive care unit under French rules: a nationwide survey of practices**

**Supporting Information**

**S1 and S2 Tables**

**S1 Table: Responses to the questionnaire regarding decisions not to re-admit a patient to the intensive care unit**

| **Question number** | **All or most of the time** | **Sometimes** | **Rarely or Never** |
| --- | --- | --- | --- |
| 3. Is the decision not to re-admit a patient to the ICU taken at the end of the patient’s initial stay in the ICU ? | 87% | 11% | 2% |
| 4. Are decisions not to readmit made using a collegial decision-making procedure in your unit? | 89% | 4% | 7% |
| 5. Are decisions not to readmit mentioned in the discharge letter? | 93% | 4% | 3% |
| 7. Is the unit that will receive the patient after discharge from the ICU informed of (without being involved in) the decision not to readmit? | 83% | 11% | 6% |
| 8. Is the unit that will receive the patient after discharge from the ICU involved in the decision not to readmit? | 30% | 47% | 23% |
| 9. Does an independent outside consultant participate (if necessary) in the decision not to readmit? | 14% | 36% | 50% |
| 10. Does the patient participate in the discussions leading to a decision not to readmit to intensive care? | 10% | 41% | 49% |
| 11. Does the patient's family and/or surrogate participate in the discussions leading to a decision not to readmit to intensive care? | 34% | 45% | 21% |
| 12 Is the patient informed about the decision not to readmit? | 28% | 40% | 32% |
| 13. Is the patient's family and/or surrogate informed about the decision not to readmit? | 72% | 19% | 9% |
| 14. Is the general practitioner or referring specialist involved in the collegial decision-making procedure? | 29% | 38% | 33% |
| 15. Is the general practitioner or referring specialist informed about the decision not to readmit? | 65% | 21% | 14% |
| 16. Are the criteria justifying the decision not to readmit noted in the patient's medical file? | 91% | 7% | 2% |
| 20. When a decision not to readmit is made, do you plan palliative care? | 41% | 45% | 14% |

**S2 Table: Percentage of responses for yes/no questions on the study questionnaire**

| **Question** | **Yes** | **No** |
| --- | --- | --- |
| 6. Are decisions not to readmit recorded in a registry in your unit? | 96% | 4% |
| 17. Are the criteria used to decide not to readmit to the ICU the same as those applied to not to admit a patient to the ICU a first time? | 61% | 39% |
| 18. If a collegial decision-making procedure is used to decide not to readmit a patient to the ICU, are there circumstances in which you may fail to respect that decision? | 96% | 4% |
| 19. Do you distinguish between a request to readmit a patient to the ICU for an intercurrent acute episode, and a request to readmit a patient for worsening of the underlying chronic disease ? | 92% | 8% |
| 21. In your opinion, is non-readmission to the ICU covered by current end-of-life legislation in France? | 91% | 9% |
