# Supplementary material for: Non-readmission decisions in the intensive care unit under French rules: A nationwide survey of practices
Source: PLoS One. 2018 Oct 18;13(10):e0205689. doi: 10.1371/journal.pone.0205689 (PMC6193659; doi:10.1371/journal.pone.0205689)
Supplement: S3 File — The completed COREQ checklist is provided in the supporting file. (DOCX) [file pone.0205689.s003.docx]

**Consolidated criteria for reporting qualitative studies (COREQ): 32-item checklist**

Developed from:

Tong A, Sainsbury P, Craig J. Consolidated criteria for reporting qualitative research (COREQ): a 32-item checklist for interviews and focus groups. *International Journal for Quality in Health Care*. 2007. Volume 19, Number 6: pp. 349 – 357

**YOU MUST PROVIDE A RESPONSE FOR ALL ITEMS. ENTER N/A IF NOT APPLICABLE**

| **No. Item** | **Guide questions/description** | **Reported on Page #** |
| --- | --- | --- |
| **Domain 1: Research team and reﬂexivity** |  |  |
| *Personal Characteristics* |  |  |
| 1. Inter viewer/facilitator | Which author/s conducted the inter view or focus group? | Not applicable, this was a survey of practices. The interviews performed to develop the questionnaire were performed by NMB. This is stated in the methods section. |
| 2. Credentials | What were the researcher’s credentials? E.g. PhD, MD | Sociologist for Nicolas Meunier-Beillard, and PhD, MD for Jean-Pierre Quenot and Jean-Philippe Rigaud. The highest degrees for each researcher are given on the title page. |
| 3. Occupation | What was their occupation at the time of the study? | Sociologist for Nicolas Meunier-Beillard, and physicians for Jean-Pierre Quenot and Jean-Philippe Rigaud. This is stated in the Methods section, |
| 4. Gender | Was the researcher male or female? | Male, this is specified in the methods sectioni. |
| 5. Experience and training | What experience or training did the researcher have? | The sociologist has a Masters degree in sociology, and the physicians have more than 5 years of experience in the ICU, and more than 10 years of third-level education. All had wide experience of qualitative research, as witnessed by their scientific publications (list available in Medline). This is stated in the methods.  Methods |
| *Relationship with participants* |  |  |
| 6. Relationship established | Was a relationship established prior to study commencement? | N/A since this was a survey of practices. |
| 7. Participant knowledge of the interviewer | What did the participants know about the researcher? e.g. personal goals, reasons for doing the research | An information letter describing the research was given to participants. Agreement to participation was to be obtained |
| 8. Interviewer characteristics | What characteristics were reported about the inter viewer/facilitator? e.g. Bias, assumptions, reasons and interests in the research topic | N/A – this was a questionnaire survey.  The assumptions behind the development of questionnaire were based on empirical interviews as outlined in the Methods section. |
| **Domain 2: study design** |  |  |
| *Theoretical framework* |  |  |
| 9. Methodological orientation and Theory | What methodological orientation was stated to underpin the study? e.g. grounded theory, discourse analysis, ethnography, phenomenology, content analysis | Methods.  The current paper presents the results to the questions in terms of frequencies, and content analysis of the free-text comments |
| *Participant selection* |  |  |
| 10. Sampling | How were participants selected? e.g. purposive, convenience, consecutive, snowball  . | Methods  The participants were selected from the physicians of several intensive care units and agreement for participation was given by all physicians. They were selected from among an electronic database of ICU physicians who had previously participated in two other studies. |
| 11. Method of approach | How were participants approached? e.g. face-to-face, telephone, mail, email | Mail, Email and telephone. |
| 12. Sample size | How many participants were in the study? | Results; 18 physicians in the qualitative part of the study and 167 patients in the quantitative part of the study. |
| 13. Non-participation | How many people refused to participate or dropped out? Reasons? | None |
| *Setting* |  |  |
| 14. Setting of data collection | Where was the data collected? e.g. home, clinic, workplace | Data collected at the hospital; Methods |
| 15. Presence of non-participants | Was anyone else present besides the participants and researchers? | No |
| 16. Description of sample | What are the important characteristics of the sample? e.g. demographic data, date | The characteristics of the respondents are given in Table 1 (Results) |
| *Data collection* |  |  |
| 17. Interview guide | Were questions, prompts, guides provided by the authors? Was it pilot tested? | The questionnaire was tested for the comprehension of each question with a panel of physicians different from those participating in the present study (Methods) |
| 18. Repeat interviews | Were repeat inter views carried out? If yes, how many? | N/A |
| 19. Audio/visual recording | Did the research use audio or visual recording to collect the data? | Audio recording (Methods) |
| 20. Field notes | Were ﬁeld notes made during and/or after the inter view or focus group? | No, since there were audio recordings |
| 21. Duration | What was the duration of the inter views or focus group? | N/A |
| 22. Data saturation | Was data saturation discussed? | Yes; Methods |
| 23. Transcripts returned | Were transcripts returned to participants for comment and/or correction? | Yes; the data was discussed using the expert panel method to develop the final questionnaire, as described in the Methods. |
| **Domain 3: analysis and ﬁndings** |  |  |
| *Data analysis* |  |  |
| 24. Number of data coders | How many data coders coded the data? | 1 for the development of the questionnaire (Methods) |
| 25. Description of the coding tree | Did authors provide a description of the coding tree? | Since the interviews were confidential, and served only as a basis for the questionnaire, this data is not freely available, but reasonably requests to the first author will be considered. |
| 26. Derivation of themes | Were themes identiﬁed in advance or derived from the data? | The themes from the questionnaires were developed from the interviews as described in the Methods |
| 27. Software | What software, if applicable, was used to manage the data? Not applicable | NVivo |
| 28. Participant checking | Did participants provide feedback on the ﬁndings? | Yes; these are described in the strengths and limitations |
| *Reporting* |  |  |
| 29. Quotations presented | Were participant quotations presented to illustrate the themes/ﬁndings? Was each quotation identiﬁed? e.g. participant number | No, since the focus of the study presented here is the questionnaire. The final questionnaire is presented as a Table in the paper. |
| 30. Data and ﬁndings consistent | Was there consistency between the data presented and the ﬁndings? | Yes (Results). |
| 31. Clarity of major themes | Were major themes clearly presented in the ﬁndings? | Yes (Results) |
| 32. Clarity of minor themes | Is there a description of diverse cases or discussion of minor themes? | Yes Discussion |

**Once you have completed this checklist, please save a copy and upload it as part of your submission. When requested to do so as part of the upload process, please select the file type: *Checklist*. You will NOT be able to proceed with submission unless the checklist has been uploaded. Please DO NOT** **include this checklist as part of the main manuscript document. It must be uploaded as a separate file.**
